# Estimating male circumcision coverage in 15 priority countries in sub‐Saharan Africa

**DOI:** 10.1002/jia2.25789

**Published:** 2021-09-21

**Authors:** Peter M. Stegman, Randy Yee, Joshua Davis, Michel Tchuenche, Rachael Linder, Lycias Zembe, Luisa Frescura, Katharine E. Kripke

**Affiliations:** ^1^ Avenir Health Glastonbury Connecticut USA; ^2^ Centers for Disease Control and Prevention Atlanta Georgia USA; ^3^ US Agency for International Development Washington DC USA; ^4^ Joint United Nations Programme on HIV/AIDS Geneva Switzerland

**Keywords:** biomedical prevention, HIV & AIDS, HIV prevention, male circumcision, sub‐Saharan Africa, UNAIDS

## Abstract

**Introduction:**

Given the importance of voluntary medical male circumcision (VMMC) in reducing HIV incidence, access to and use of quality data for programme planning and management are essential. Unfortunately, such data are currently not standardized for reliable and consistent programme use in priority countries. To redress this, the UNAIDS Reference Group (RG) on Estimates, Modelling, and Projection worked with partner Avenir Health to use the Decision Makers Program Planning Toolkit (DMPPT) 2 Online to provide estimates of VMMC coverage and to support countries to set age‐ and geographic‐specific targets. This article describes the methods and tools used for assembling, reviewing and validating VMMC programme data as part of the 2021 Estimates process.

**Discussion:**

The approach outlined for integrating VMMC data using the DMPPT2 Online required significant country engagement as well as upgrades to the DMPPT2 Online. The process brought together local‐level VMMC stakeholders, for example Ministries of Health, the Joint United Nations Programme on HIV/AIDS (UNAIDS), the US President's Emergency Plan for AIDS Relief, the World Health Organization (WHO), VMMC implementers and so on, to review, amend and agree on historical and more recent VMMC data. The DMPPT2 Online was upgraded to align with the Spectrum and Naomi models used in the Annual HIV Estimates process. In addition, new and revised inputs were incorporated to enhance accuracy of modelled outputs. The process was successful in mobilizing stakeholders behind efforts to integrate VMMC into the annual HIV Estimates process and generating comprehensive, country‐owned and validated VMMC data that will enhance programme monitoring and planning.

**Conclusions:**

VMMC programme data from most of the priority countries were successfully reviewed, updated, validated and incorporated into the annual HIV Estimates process in 2020. It is important to ensure that these data continue to be used for programme planning and management. Current and future data issues will need to be addressed, and countries will need ongoing support to do so. The integration of the DMPPT2 Online into the annual HIV Estimates process is a positive step forward in terms of streamlining country‐owned planning and analytical practices for the HIV response.

## 1 INTRODUCTION

After three randomized controlledtrials demonstrated that male circumcision (MC) reduces the risk of human immunodeficiency virus (HIV) acquisition by approximately 60% [1, 2, 3], the Joint United Nations Programme on HIV/AIDS (UNAIDS) and the World Health Organization (WHO) recommended in 2007 that countries with generalized epidemics and low MC prevalence establish and scale up voluntary medical male circumcision (VMMC) as a component of comprehensive HIV prevention [4]. Since then, 15 countries in sub‐Saharan Africa prioritized by UNAIDS and WHO have included VMMC as part of prevention programming: Botswana, Eswatini, Ethiopia, Kenya, Lesotho, Malawi, Mozambique, Namibia, Rwanda, South Africa, South Sudan, Tanzania, Uganda, Zambia and Zimbabwe.

Building from this, UNAIDS and WHO developed the Joint Strategic Action Framework to Accelerate the Scale‐up of VMMC for HIV Prevention in eastern and southern Africa, 2012–2016, to guide scaling up VMMC among men aged 15–49 years [5]. This was followed in 2016 by the Framework for Voluntary Medical Male Circumcision: Effective HIV Prevention and a Gateway to Adolescent Boys’ and Men's Health in eastern and southern Africa by 2021, to help integrate VMMC into sexual and reproductive health services for boys and men [6, 7]. Currently, it is estimated that around 26 million VMMCs have been performed in the 15 priority countries. Despite this, UNAIDS Fast‐Track targets for VMMC have yet to be achieved [6]. To accelerate progress, programmes need accurate and reliable data for programme monitoring, strategic planning and effective resource allocation for VMMC.

Unfortunately, such data are currently not standardized for reliable and consistent programme use in any of the priority countries. To redress this, the UNAIDS Reference Group (RG) on Estimates, Modelling and Projections began working with Avenir Health and other partners in July 2020 to use the Decision Makers Program Planning Toolkit (DMPPT) 2 Online to provide estimates of VMMC coverage and to support countries to set age‐ and geographic‐specific targets. The objective of this process was to better integrate VMMC into the UNAIDS HIV estimates process, which currently uses the Spectrum/Estimation and Projection Package, a statistical model that estimates national‐level HIV prevalence and incidence using surveillance, surveys and programme data [8], and Naomi, a model that produces district‐level estimates of HIV prevalence, incidence and ART coverage [9]. Integration of VMMC into the estimates process also contributes to the ongoing work of UNAIDS and Avenir Health to assist countries to own and sustain their own data systems for HIV/AIDS. This article describes the methods and tools used in assembling, reviewing and validating VMMC programme data as part of the 2021 estimates process.

## 2 DISCUSSION

### 2.1 The value of the DMPPT

Through various iterations, the DMPPT has a long association with VMMC. In 2009, Avenir Health (formerly Futures Institute) developed the DMPPT to examine and estimate the epidemiological impacts and cost‐effectiveness of scaling up VMMC in countries with high HIV prevalence and low levels of circumcision [10, 11, 12]. This was followed by the DMPPT2, developed in 2013, to answer questions about the impact and cost‐effectiveness of VMMC targeting specific age groups and sub‐national geographies. Described in detail elsewhere [13], DMPPT2 modelling analyses have formed the basis for programme planning over the years in numerous VMMC priority countries [14] and informed global guidance from the US President's Emergency Plan for AIDS Relief (PEPFAR) and UNAIDS [15]. The model does have limitations such as the assumption that practices that resulted in the levels of MC prevalence prior to the start of the programme do not change after the introduction of VMMC [16]. Nevertheless, its contribution to VMMC over the years has been important. Today, the DMPPT2 is online, allowing users to log in and generate age‐ and district‐level specific coverage estimates, numerical targets and impact projections. The value in having a user‐friendly online tool is to put VMMC programme personnel in a position to better manage, analyse and deploy their data for planning and policy decision making.

### 2.2 Approaches to updating the DMPPT2 Online

The UNAIDS RG worked with Avenir Health and other reference group organizations, including the WHO, US Agency for International Development (USAID), Centers for Disease Control and Prevention (CDC), the Bill and Melinda Gates Foundation and Imperial College London, to develop an approach to integrating the DMPPT2 Online into the annual HIV estimates process. Setting up or updating the DMPPT2 Online models for each priority country required significant work to populate the tool and guide each country through the process. An initial step was to update or collect, validate and input VMMC programme data (collected August 2020–March 2021) into the DMPPT2 Online.

#### 2.2.1 Country engagement

Working through the UNAIDS Strategic Information Advisors in VMMC priority countries, teams were established to populate DMPPT2 Online data collection templates. They were generally composed of VMMC programme staff from the health ministry, UNAIDS, PEPFAR counterparts and implementing partners. Discussions with countries began with two large‐scale webinars in August 2020, hosted by UNAIDS. These were intended to inform VMMC stakeholders about the rationale and objective of using the DMPPT2 Online to integrate VMMC into the annual HIV estimates process. Thereafter, individual country teams, whether already using the DMPPT2 Online or not, were provided with ongoing technical assistance from Avenir Health through, among others, virtual conference calls, telephone calls and targeted emails. Country teams were supported, as far as possible, to build their own capacities to work through their VMMC programme data, validate them and populate the DMPPT2 Online data collection template.

#### 2.2.2 Upgrades to the DMPPT2 Online

To integrate the DMPPT2 Online more easily into the UNAIDS annual HIV estimates process and facilitate trouble‐free access and use by VMMC priority countries, several updates were made. For the DMPPT2 Online to align with other models used in the estimates process (i.e. Spectrum/EPP and Naomi), each country's sub‐national units (SNUs) and their corresponding population sizes were unified with the standard SNU nomenclature and population estimates of the Naomi model.

Baseline circumcision prevalence for the model was established using country Demographic and Health Surveys or AIDS Impact Surveys from before VMMC programme initiation. Adding programme data into the model allows countries to set age‐ and SNU‐specific targets, project coverage increases, impact and unmet need. In 2020, model inputs were improved using the work of Cork et al. [17], who applied a Bayesian geostatistical model to analyse MC prevalence data from household surveys in 38 countries in sub‐Saharan Africa. They provided revised district‐level baseline estimates that were incorporated into the data collection template to enable the DMPPT2 Online to generate more accurate projections of VMMC coverage for planning and monitoring.

**Figure 1:**
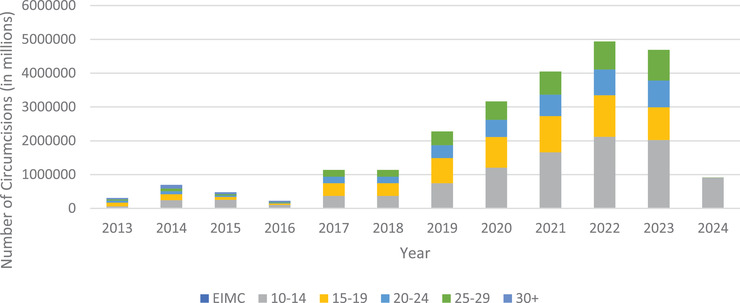
Number of circumcisions performed through 2018 (fabricated example historical data from a fictitious country) and those needing to be done (by age group and year) starting in 2019 to meet a user‐defined target of 80% coverage among 10‐ to 29‐year‐olds by 2024 (Source: DMPPT2 Online). EMIC, Early Infant Male Circumcision

Figure 1 displays a common output of the DMPPT2 Online. Using fabricated example data, the model estimates coverage by adding baseline circumcisions to VMMC programme data, taking client age progression and mortality into account. The model also projects the number of circumcisions that would be necessary to meet a user‐defined target scenario of 80% coverage for the age groups 10–14, 15–19, 20–24 and 25–29 by 2024. In this example, the model demonstrates a scale‐up strategy that aggressively targets younger age groups to effectively reduce the number of men in older age groups that will require circumcision in later years.

Additional updates to the DMPPT2 Online include the ability to analyse VMMC data and project targets according to a user‐specified fiscal year (e.g. government, United Nations, PEPFAR, etc.) by inputting VMMC programme data by quarter. With this modification, the tool correctly projects coverage estimates and targets by the indicated fiscal year starting in 2020. Once the DMPPT2 Online data collection templates were populated and agreed by country VMMC stakeholders, they were uploaded to the UNAIDS AIDS Data Repository, which acts as a central data exchange for improving the accessibility and consistency of HIV data globally.

**Figure 2:**
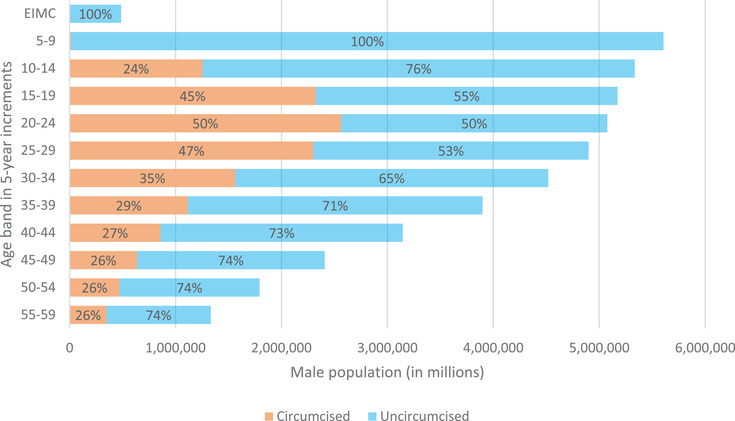
Circumcised and uncircumcised as a proportion of total population of males in each age group in 2021 (fabricated example data) (Source: DMPPT2 Online).

The DMPPT2 Online was also modified to export results that could be read by Spectrum for output to the PEPFAR Country Operational Plan (COP) data pack indicators, namely: number of men circumcised during the reporting period and number of men ever circumcised, by 5‐year age group. Figure 2 is another output from the model and uses the same fabricated example data as before to show the unmet need for VMMC in 2021. It presents total male population for each age band and the percentage circumcised or not. According to the data, the largest circumcised populations are in the 15‐ to 19‐, 20‐ to 24‐, and 25‐ to 29‐year age bands. In this hypothetical example, no infants or boys under age 10 have been circumcised. Few VMMC priority countries set targets for EIMC, and VMMC of boys between the ages of 60 days and 10 years is not generally promoted due to the increased risk of adverse events. However, these age groups are included in the programme data inputs and the calculations of the tool.

#### 2.2.3 Additional support for DMPPT2 Online data updates

Members of the PEPFAR Interagency Collaborative for Program Improvement developed a data validation tool to help country teams identify anomalies or gaps in their VMMC programme data. With this tool, teams could focus their reviews and make quick corrections to move the entire process forward. This was especially important for countries that had little to no exposure to the DMPPT2 Online. The Excel‐based data validation tool automatically extracted VMMC data from the populated DMPPT2 Online data collection template and produced a series of tables and visualizations presenting year‐on‐year trends by age and total numbers of VMMCs performed, and the percent increase or decrease in these indicators. Trends could be viewed either nationally or by specific SNU, and by any defined series of dates. Based on the parameters set by the user, SNUs could be ranked from the highest to lowest standard deviation to allow country teams to easily identify and isolate areas of possible concern and to focus efforts where review of data quality appeared to be most critical. However, despite the generally user‐friendly format of the data validation tool, few country teams employed it as part of their VMMC data update and validation process. In general, teams suggested that the use of the validation tool became an additional, secondary process, the objectives of which were already being met through the VMMC programme data reviews they were undertaking.

### 2.3 Results of the process

In this initial exercise, 13 of the 15 VMMC priority countries completed the data assessment, update and validation, and uploaded their data to the DMPPT2 Online. Two countries, South Africa and South Sudan, opted out of the process for the current round of HIV estimates and COP development. South Africa has other tools and resources it uses for managing its VMMC programme data for planning. At present, the government and its implementing partners are satisfied to move ahead with those, rather than update their inputs to the DMPPT2 Online. South Sudan's VMMC programme has been limited. After a series of consultations, the country decided that they lacked the necessary data inputs to make use of the tool at this time. Both countries continue to report their national‐level age‐disaggregated numbers of circumcisions to the Global AIDS Monitoring system, and both countries will have the opportunity to prepare their data and utilize the DMPPT2 Online for annual planning should they elect to do so.

Through this process, a number of benefits for VMMC programming globally have been realised. Firstly, the DMPPT2 Online has served as a mechanism through which VMMC can be better integrated into the UNAIDS annual HIV estimates process. Secondly, by embarking on this process, programmes in priority countries have arrived at a VMMC dataset that is comprehensive, validated and agreed to enhance programme monitoring and target setting. This positive development is evidenced by the fact that all VMMC stakeholders came together to provide and review all available VMMC data (historical and recent) to produce a nationally representative picture of the programme. Thirdly, the outputs of the DMPPT2 Online have been enhanced and made more reliable and robust with the VMMC data revision process and updated inputs driving greater accuracy in the model.

The idiosyncratic and complex nature of different country programmes raised a few challenges that will need to be addressed to ensure the most accurate VMMC data are available for programme planning and monitoring. These have been discussed in more detail elsewhere [16]. Because the DMPPT 2 Online is a demographic model that assesses coverage of MC at the district level, it is critical that the numerator (number of circumcisions conducted – usually recorded in databases according to the location of the circumcision site) and the denominator (size of the eligible population – derived from Census estimates) match. Three specific instances have been identified where this can challenge accurate coverage estimates and, hence, appropriate programme planning:

- *Short‐term and service‐related migration*. Male populations often move about within a country, for economic, political or other reasons. When they are circumcised in an SNU other than where they would normally be counted in the census, this falsely inflates numbers in one SNU at the expense of the other, sometimes leading to VMMC coverage estimates higher than 100% in some age groups in some SNUs [8].
- *Military personnel circumcisions*. In several countries, the US Department of Defense (DoD) is a programme implementation partner under PEPFAR. Circumcision numbers under this initiative are generally, but not always, reflected in national programme data reporting. However, data attributing DoD‐supported circumcisions to the specific SNUs where each military client (and their family members) resides, as per the census, either are not collected or access is strictly limited. This artificially increases circumcision numbers in the localities where the circumcision takes place and decreased them in the locality where the client comes from, giving an inaccurate picture of MC coverage in each location. To date, there is no agreed and workable solution for adjusting/properly accounting for the military personnel circumcisions in any of the countries where this is an issue.
- *Refugee populations*. Circumcisions performed among refugee populations may be included in national VMMC programme data. However, obtaining and applying these data can be problematic and may falsely inflate overall coverage levels if the refugees are not included in the Census‐based population estimates. If there are estimates of the refugee population size and its baseline MC prevalence, refugees can be included in the DMPPT2 Online as a separate SNU or population.

## 3 CONCLUSIONS

VMMC programme data from most of the priority countries were successfully cleaned and validated in order to be incorporated into the annual HIV estimates process. For these countries, future updates of their annual programme accomplishments will require less intensive work. The DMPPT2 Online can assist with ensuring that these data are owned by countries and continue to be used for programme planning and management. Further, use of the model will help countries address current and future data issues. As national VMMC programmes continue to mature, there will be need for processes and tools to help make sense of trends, and awareness of future trajectories may provide important guidance to national programmes. The integration of the DMPPT2 Online into the annual HIV estimates process is a positive step forward in terms of streamlining existing planning and analytical practices for the HIV response.

## FUNDING

This work was funded through the USAID supported Project SOAR (Supporting Operational AIDS Research) and UNAIDS.

## COMPETING INTERESTS

The authors declare that they have no competing interests.

## AUTHORS' CONTRIBUTIONS

PS and RY wrote and prepared the original draft. PS, RY, KK, JD, MT, RL, LZ and LF reviewed and edited the draft. PS finalized writing of the manuscript.
